# Effects of formal aquatic activities on health and developmental outcomes in infants and toddlers younger than 36 months: a systematic review and meta-analysis

**DOI:** 10.3389/fped.2026.1910257

**Published:** 2026-09-30

**Authors:** Jiayi Lyu, Guangnan Liu, Yang Liu, Lei Lei, Xinyi Li, Mo Bai, Leyi Sun, Jie Gao

**Affiliations:** 1 School of Education, Beijing Sport University, Beijing, China; 2 Department of Ophthalmology, Peking Union Medical College Hospital, Chinese Academy of Medical Sciences & Peking Union Medical College, Beijing, China; 3 Beijing No. 24 Middle School, Beijing, China; 4 Sports Coaching College, Beijing Sport University, Beijing, China

**Keywords:** aquatic activities, hydrotherapy, infant swimming, infants, systematic review

## Abstract

**Aims:**

To systematically review and meta-analyze controlled evidence on the effects of formal aquatic activities on health and developmental outcomes in infants and toddlers younger than 36 months.

**Background:**

Formal aquatic activities—including infant swimming, neonatal swimming, hydrotherapy, and warm-water bathing—are increasingly used in early-life care, yet existing studies vary in populations, intervention formats, and outcome measures, making overall effects difficult to determine. A structured synthesis is needed to clarify their potential role in early health and development.

**Methods:**

We conducted an updated search of PubMed, Ovid MEDLINE(R) ALL, EMBASE, PsycINFO, Scopus, SportDiscus, Web of Science, and EBSCOhost (December 2022 to May 25, 2026) and CNKI (inception to May 25, 2026). Controlled studies evaluating formal aquatic activities in infants or toddlers younger than 36 months were eligible. Outcomes were coded and organized using the WHO International Classification of Functioning, Disability and Health for Children and Youth (ICF-CY) framework. Random-effects meta-analyses pooled standardized mean differences (SMDs) for continuous outcomes and risk ratios (RRs) for binary developmental classification outcomes. Sensitivity and subgroup analyses were conducted where heterogeneity was substantial.

**Results:**

25 studies (3,526 participants; 1,822 aquatic intervention, 1,704 comparator) were included; 22 contributed to the meta-analysis. Across six outcome domains, 87 effect sizes were synthesized. Aquatic activities showed favourable effects on: neurodevelopmental and motor function (SMD = 0.96, 95% CI 0.78–1.15), growth and anthropometry (SMD = 0.54, 95% CI 0.44–0.63), physiological regulation and safety (SMD = 0.85, 95% CI 0.49–1.22), beneficial developmental classification (RR = 1.45, 95% CI 1.29–1.62), jaundice burden and bilirubin metabolism (SMD = 2.31, 95% CI 1.37–3.26), and feeding, stool transition, and digestion (SMD = 0.81, 95% CI 0.61–1.01). Heterogeneity was highest for jaundice-related outcomes; sensitivity and subgroup analyses confirmed the favourable direction of effects.

**Conclusions:**

This ICF-CY-informed systematic review and meta-analysis suggests that formal aquatic activities may have beneficial effects across multiple health and developmental domains in infants and toddlers under 36 months. These findings support using aquatic activities as a complementary approach in early-life care, while underscoring the need for more rigorous randomized trials, standardized intervention protocols, and longer follow-up to strengthen the evidence base.

## Introduction

The first three years of life constitute a critical window for neuromotor, cognitive, and physiological development (1, 2). Early interventions that provide structured sensory, motor, and caregiver supported experiences may influence developmental trajectories and short-term health outcomes (3). Among these interventions, formal aquatic activities have attracted increasing attention in early childhood, clinical, rehabilitation, day care, and family settings (4). These activities are not limited to conventional infant swimming, but may also include neonatal swimming, parent infant aquatic exploration, hydrotherapy, aquatic therapy, and infant spa programs that combine water-based movement with other forms of sensory stimulation (4). By using the distinctive properties of water, including buoyancy, tactile and vestibular stimulation, and reduced gravitational loading, these interventions may facilitate active movement, promote motor coordination, and support physiological regulation (5).

However, the effects and safety of formal aquatic activities in infants and toddlers remain incompletely established. Previous literature has suggested potential benefits for early motor performance and developmental outcomes, but findings have not been fully consistent, and some observed effects may be influenced by maturation, baseline health status, parental factors, or differences in intervention exposure. In addition, concerns have been raised regarding possible health and safety related outcomes, including gastrointestinal infection, skin irritation, respiratory symptoms, physiological stability, and drowning prevention (4, 6). Existing evidence is also limited by the small number of eligible studies focused specifically on children younger than 36 months, the predominance of observational or quasi experimental designs, small intervention samples in several studies, limited control of confounding factors, and the absence of evidence from natural water environments. These limitations indicate that the current evidence base requires cautious interpretation and more structured synthesis (6, 7). The heterogeneity of outcomes reported in primary studies further complicates evidence synthesis and limits the interpretability of pooled findings. Without a pre-defined conceptual framework, meta-analytic results may be shaped primarily by data availability rather than by underlying developmental, physiological, or clinical rationale (4, 8).

To address this challenge, the present review adopts the World Health Organization's International Classification of Functioning, Disability and Health for Children and Youth framework to classify extracted outcomes into six pre specified domains: neurodevelopmental and motor function, growth and anthropometry, jaundice burden and bilirubin metabolism, feeding, stool transition and digestion, physiological regulation and safety, and beneficial developmental classification (9). This structured approach ensures that outcomes are aggregated according to conceptual similarity and clinical relevance rather than *post hoc* statistical convenience.

The objective of this systematic review and meta-analysis is therefore to evaluate the effects of formal aquatic activities on health and developmental outcomes in infants and toddlers younger than 36 months. Guided by a pre-defined ICF CY framework, this review aims to enhance the transparency, comparability, and interpretability of evidence on aquatic interventions during early life.

## Methods

### Study design

This systematic review and meta-analysis synthesized evidence from controlled studies evaluating the effects of formal aquatic activities on health and developmental outcomes in infants and toddlers younger than 36 months. The review protocol was registered in PROSPERO (CRD420261404287). In the design and reporting of this review, we followed the Preferred Reporting Items for Systematic Reviews and Meta-Analyses checklist.

### Search methods for the identification of studies

#### Information sources and search strategy

A comprehensive literature search was conducted in PubMed, Ovid MEDLINE(R) ALL, EMBASE, PsycINFO, Scopus, SportDiscus, Web of Science, EBSCOhost and CNKI to identify studies evaluating formal aquatic activities in infants and toddlers younger than 36 months. The complete search strategy for each database is provided in Supplementary Material 1. Search terms were developed using a combination of free-text words and database-specific controlled vocabulary, where applicable. The search strategy was structured around three core concepts: aquatic exposure, infancy or toddlerhood, and controlled intervention evidence. Terms related to aquatic exposure included infant swimming, neonatal swimming, baby swimming, hydrotherapy, aquatic therapy, hydrokinesiotherapy and warm-water bathing. Terms related to the population captured infants, neonates, newborns and toddlers. The third concept was designed to identify controlled or comparative intervention studies. The search period for the international databases was selected to update the evidence base established by Santos et al. (2023) (4), whose systematic review covered the relevant literature through December 2022. Accordingly, PubMed, Ovid MEDLINE(R) ALL, EMBASE, PsycINFO, Scopus, SportDiscus, Web of Science and EBSCOhost were searched from December 2022 to May 25, 2026. CNKI was searched from database inception to May 25, 2026 because it was not covered by the previous review.

#### Searching for other sources

To ensure continuity with the evidence base preceding the updated database search, we manually checked potentially relevant records identified in the review by Santos et al. (2023) (4), as documented in the study flow diagram. In addition, the reference lists of all studies included in the present review were manually screened to identify potentially eligible studies not captured through the electronic database searches. Articles identified through these supplementary approaches were retrieved in full text and reassessed using the same eligibility criteria as records identified through the updated database searches. Studies were considered eligible for the systematic review only when they evaluated a formal aquatic activity in infants or toddlers younger than 36 months using a controlled design.

### Eligibility criteria

Eligible studies were selected according to the PICOS framework. No restrictions were applied to country or region of publication. Full-text articles published in English or Chinese were considered eligible. Duplicate publications, conference abstracts without sufficient methodological information, study protocols without outcome data, case reports, uncontrolled case series, single-group pre-post studies, cross-sectional studies and reports without extractable controlled outcome data were excluded.

#### Population

Eligible participants were infants and toddlers younger than 36 months. This included healthy term infants, preterm infants, hospitalized neonates, infants or neonates with developmental delay, and neonates with clinical conditions such as jaundice, hyperbilirubinaemia, bilirubin encephalopathy, hypoxic-ischaemic encephalopathy, physiological weight loss or scleredema. Studies including children outside this age range were eligible only when data for participants younger than 36 months could be extracted separately.

#### Intervention

The intervention of interest was participation in a formal aquatic activity. Formal aquatic activities included structured infant swimming, neonatal swimming, baby swimming, swimming exercises, hydrotherapy, aquatic therapy, hydrokinesiotherapy and warm-water bathing delivered in clinical, rehabilitation, daycare, hospital or family-centred settings. When aquatic exposure was combined with touch, massage, kangaroo care, rehabilitation, routine neonatal care or other cointerventions, the intervention was treated as an aquatic activity package rather than isolated water exposure.

#### Comparator

Eligible studies were required to include a comparator group that did not receive the formal aquatic activity under investigation. Comparators could include routine care, standard neonatal care, usual rehabilitation without aquatic exposure, non-aquatic nursing care, usual family-centred care or other non-aquatic intervention conditions. Studies were excluded if the comparator group received the same aquatic intervention as the intervention group during the analysed period.

#### Outcome measures

Eligible outcomes were health or developmental outcomes relevant to infants and toddlers and classifiable within the ICF-CY framework. These outcomes included neurodevelopmental and motor function, growth and anthropometry, jaundice burden and bilirubin metabolism, feeding, stool transition and digestion, physiological regulation and safety, and beneficial developmental classification. For quantitative synthesis, continuous outcomes were required to provide sufficient data to calculate or extract standardised mean differences, and binary developmental classification outcomes were required to provide sufficient event and sample-size data to calculate risk ratios.

#### Study design

Eligible studies used a controlled intervention design with at least one formal aquatic activity group and one comparator group. Randomised controlled trials, randomised controlled intervention studies, non-randomised controlled intervention studies, sequentially allocated controlled studies, quasi-experimental controlled studies and controlled pilot studies were considered eligible. Studies were excluded if they did not include a comparator group, did not evaluate a formal aquatic activity, or did not provide extractable controlled outcome data.

### Data collection and analysis

#### Selection of studies

After duplicate records had been removed (Y.L. and L.S.), two reviewers (J.L. and G.L.) independently screened the titles and abstracts together as a single initial screening stage against the predefined eligibility criteria. A separate title-only screening stage was not performed. Full texts were retrieved for records that were potentially eligible or for which eligibility could not be determined from the title and abstract information, with retrieval assistance from L.L. and X.L. This combined title-and-abstract approach is consistent with established systematic-review guidance describing initial record screening at the title-and-abstract level before full-text assessment (10, 11). The same two reviewers (J.L. and G.L.) then independently assessed the full-text articles. Disagreements at any stage were resolved through discussion with a senior reviewer (J.G.). Reasons for exclusion at the full-text stage were recorded and grouped as intervention mismatch, outcome mismatch, absence of extractable controlled data or other reasons, independently verified by M.B., and documented in the study flow diagram. Studies reporting only medians, non-parametric summaries or insufficient quantitative data were retained for descriptive synthesis when they met the eligibility criteria but could not contribute to pooled estimates.

#### Data extraction and management

Data extraction was performed independently by two reviewers (J.L. and G.L.) using a standardized Microsoft Excel form. Extracted information included study identifier, author and year, country or region, study design, population characteristics, health status, intervention and comparator sample sizes, intervention description, comparator condition, type of aquatic exposure, cointerventions, primary outcomes, age or gestational-age description, sex distribution, meta-analysis readiness and final synthesis domain. Extracted data were cross-checked by two additional reviewers (L.L. and Y.L.). Discrepancies were checked against the original articles and resolved by discussion; unresolved disagreements were adjudicated by a senior reviewer (J.G.).

For continuous outcomes, effect sizes were extracted or, when not directly reported, calculated as standardized mean differences using R software. When lower raw values indicated a more favourable clinical status, such as bilirubin concentration, jaundice index, jaundice regression time, time to first defecation or stool-transition timing, the direction of effect was reversed so that positive values consistently favoured the aquatic intervention. For binary developmental classification outcomes, risk ratios greater than 1 indicated a higher probability of beneficial developmental classification in the aquatic-intervention group.

#### Data analysis

Categorical variables were summarized as counts and percentages. Continuous study-level characteristics were summarized using medians and interquartile ranges when appropriate. Continuous outcomes were synthesized as standardized mean differences, whereas binary developmental classification outcomes were synthesized as risk ratios. All effect-size calculations and statistical analyses were performed using R software (version 4.6.0; R Foundation for Statistical Computing, Vienna, Austria), primarily with the meta package (version 8.5-0) and the metafor package (version 5.0-1). Statistical analyses were primarily conducted by J.L. and G.L., with independent verification performed by X.L. and M.B.

#### Risk of bias assessment

Risk of bias was assessed using the original Cochrane Risk of Bias tool (RoB 1). The assessment followed the established RoB 1 domain-based framework, with each domain judged as having low, unclear, or high risk of bias. Two reviewers (J.L. and G.L.) independently assessed each included study across the following domains: random sequence generation, allocation concealment, blinding of participants and personnel, blinding of outcome assessment, incomplete outcome data, selective reporting and other bias. Assessments were independently verified by L.L. and L.S. Each domain was judged as low, unclear or high risk of bias, as appropriate. Any disagreement was discussed with a senior reviewer (J.G.) until consensus was reached. The final judgements were summarized using a domain-level risk-of-bias graph and a study-level traffic-light plot.

### Data synthesis

#### Data pre-processing

Before meta-analysis, all extracted outcomes were coded and reorganized according to the WHO International Classification of Functioning, Disability and Health for Children and Youth framework. Two reviewers (J.L. and G.L.) independently mapped each outcome to the most relevant ICF-CY category using the original outcome wording, measurement instrument and clinical meaning reported in the included studies. The ICF-CY coding was subsequently cross-checked by five additional reviewers (Y.L., L.L., X.L., M.B., and L.S.). Disagreements were resolved through discussion with the review lead (J.G.). Detailed procedures for ICF-CY coding and outcome-domain construction are provided in Supplementary Material 1.

To improve interpretability and avoid data-driven outcome grouping, the coded outcomes were further organized into six analysis-ready domains before pooled-effect computation: neurodevelopmental and motor function; growth and anthropometry; jaundice burden and bilirubin metabolism; feeding, stool transition and digestion; physiological regulation and safety; and beneficial developmental classification. Outcomes were not assigned to these domains solely because statistical data were available; they also had to be conceptually consistent with the predefined ICF-CY-informed domain structure.

Four aggregation principles were applied. First, outcomes were grouped when they belonged to the same ICF-CY chapter and reflected the same physiological or developmental system. Second, standardized developmental assessment outcomes were combined when the instruments assessed cognitive, neuromotor and mobility-related abilities as integrated developmental constructs. Third, physiological outcomes crossing different ICF-CY chapters were grouped only when neonatal clinical practice treated them as part of the same regulation or safety construct. Fourth, binary developmental screening outcomes were synthesized together when they addressed the same classification question, namely whether an infant or toddler was classified as developmentally on track.

#### Meta-analysis

Random-effects meta-analyses were fitted separately within each pre-specified outcome domain using the meta and metafor packages in R. Continuous outcomes were pooled as standardized mean differences (SMDs), whereas binary beneficial developmental classification outcomes were synthesized as risk ratios (RRs). For binary outcomes, RRs were analysed on the logarithmic scale and back-transformed for presentation. The number of contributing studies and extracted effect sizes was reported for each domain to make within-study multiplicity transparent. Non-randomized studies were included alongside randomized trials because all included studies employed a controlled intervention design with a predefined comparator group, and risk of bias was assessed uniformly across designs.

Between-study heterogeneity was assessed using the *I*^2^ statistic, *τ*^2^ and Cochran's *Q* test. The *I*^2^ statistic was used to describe the proportion of total variability attributable to heterogeneity rather than sampling error, whereas *τ*^2^ represented the estimated between-study variance. A Cochran's *Q* test *P* value less than.05 was considered to indicate statistically detectable heterogeneity. When substantial heterogeneity was observed, potential sources of variation were examined in relation to population characteristics, intervention components and outcome definitions.

Funnel plots were generated for each outcome domain to visually assess small-study patterns and potential publication bias. Leave-one-out sensitivity analyses were performed when *I*^2^ was greater than or equal to 50%, by sequentially removing one study at a time and recalculating the pooled estimate. Because the jaundice burden and bilirubin metabolism domain showed substantial heterogeneity, subgroup analyses were further conducted for this domain to explore whether the pooled effect differed across clinically relevant study or intervention characteristics.

## Results

The study selection process is summarized in Figure 1. Database searches yielded 10,255 records; after removal of 5,444 duplicate records, 4,811 records entered the combined title-and-abstract screening stage, 1,135 records were retained for further eligibility assessment, of which 179 were subsequently assessed in full text. An additional 18 full-text articles were identified from Santos et al. (2023) and assessed using the same eligibility criteria. At the full-text stage, articles were excluded mainly because the intervention did not meet the study definition, the outcomes were outside the scope of this review, or extractable controlled outcome data were unavailable. Fourteen of the additional articles identified from Santos et al. (2023) also lacked extractable controlled data. Overall, 25 studies met the eligibility criteria and were included in the review, of which 22 were included in the meta-analysis.

**Figure 1:**
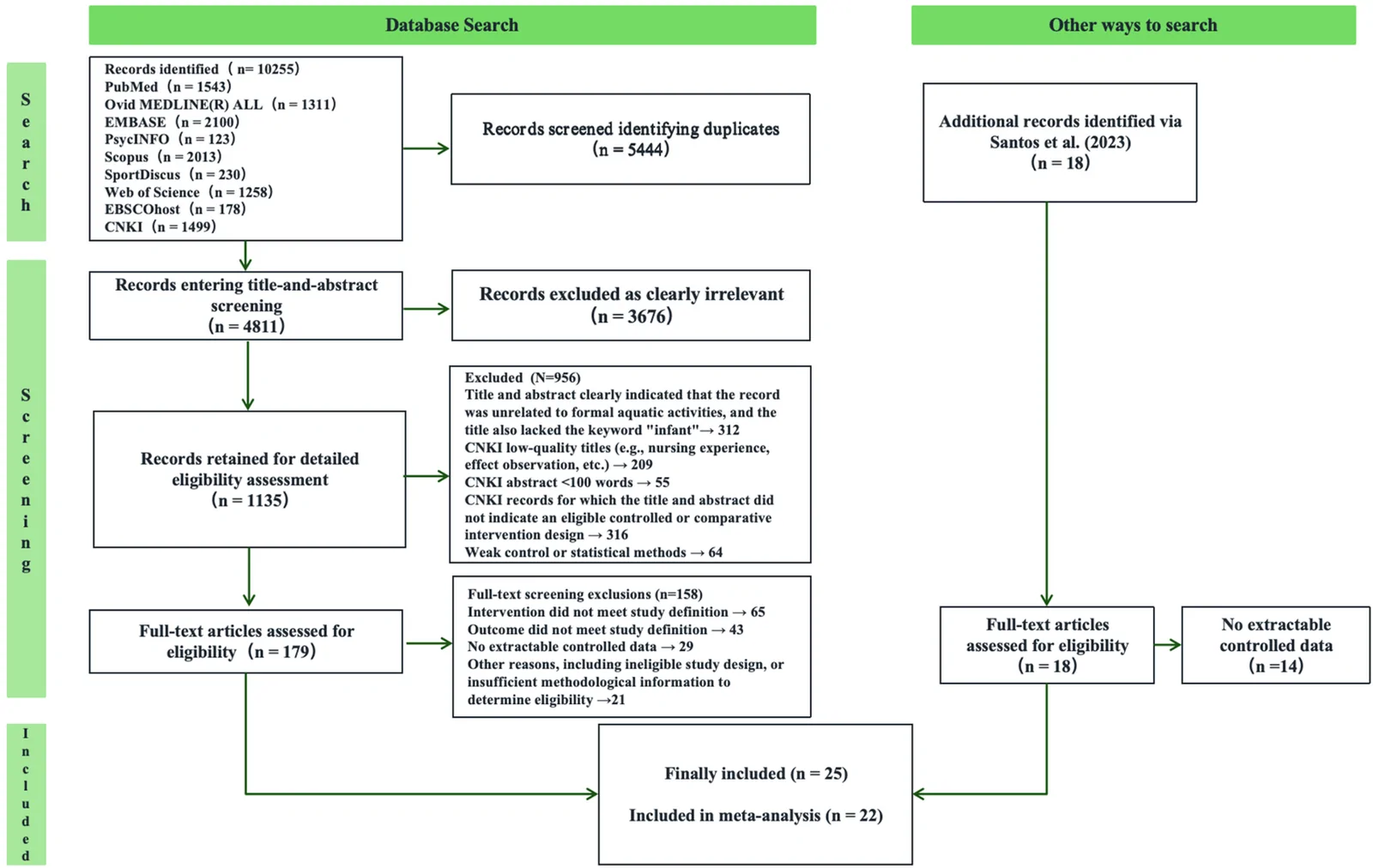
Preferred reporting items for systematic reviews and meta-analyses flow chart of included studies.

### Study and intervention characteristics

According to Table 1, the final synthesis included 25 controlled intervention studies involving 3,526 infants and toddlers, with 1,822 participants in the aquatic-intervention groups and 1,704 in the comparator groups. Most studies were conducted in China (19/25), followed by Brazil (4/25), Italy (1/25) and the United States (1/25). Some studies recruited healthy full-term or normal newborns, whereas others focused on hospitalized or preterm newborns, infants and toddlers with delayed functional mobility or neurological developmental delay, and neonates with specific clinical conditions, including jaundice, hyperbilirubinemia, bilirubin encephalopathy, hypoxic-ischemic encephalopathy, physiological weight loss and scleredema.

**Table 1:** Study characteristics.

Study	Country	Population	Design	n (I/C)	Aquatic intervention	Main six-domain outcomes	Main original outcome measures and assessment methods
Leo et al. 2022 (12)	Italy	Healthy full-term infants; post-intervention analytic sample *n* = 28	Non-randomized controlled pilot study	14/14	Baby swimming/aquatic group	Neurodevelopmental and motor function	PDMS-2: reflexes, stationary position, locomotion, grasping, visual-motor integration, gross-motor quotient, fine-motor quotient, and total-motor quotient.
Zhao et al. 2005 (18)	China	Normal term newborns during birth hospitalization	Controlled intervention study; sequential allocation described	223/ 154	Neonatal swimming twice daily during hospitalization	Growth and anthropometry	Discharge weight measured before discharge, analysed separately for vaginal- and cesarean-delivery subgroups (kg).
McManus and Kotelchuck 2007 (13)	United States	Infants and toddlers aged 6 to 30 months with delayed functional mobility	Non-randomized controlled intervention study	15/22	Weekly aquatic therapy plus home-based early intervention for 36 weeks	Neurodevelopmental and motor function	Age-adjusted normalized change score on the Gross Motor Subscale of the Mullen Scales of Early Learning from baseline to 36 weeks.
Araujo et al. 2023 (28)	Brazil	Babies aged 4 to 18 months attending daycare centers	Quasi-experimental controlled intervention study	24/37	Family-centered KITE aquatic environment sessions twice weekly for 4 weeks	Beneficial developmental classification	Typical overall NPMD classification, typical AIMS classification, and typical Denver II classification at post-intervention.
Franca et al. 2026 (19)	Brazil	Hospitalized preterm newborns with poor repertoire general movements	Randomized controlled trial	17/17	Six alternate-day 10-minute bucket hydrokinesiotherapy sessions	Growth and anthropometry	Body weight at the end of the intervention measured using an electronic infant scale (g).
Tedesco et al. 2022 (20)	Brazil	Hospitalized stable preterm newborns with low birth weight	Randomized controlled trial	17/17	Two 10-minute bucket hydrokinesiotherapy sessions on alternate days	Growth and anthropometry; Physiological regulation and safety	Body-weight gain between protocol days measured using an electronic infant scale (g); oxygen saturation on day 1 post-intervention measured by pulse oximetry (%).
Feng 2025 (30)	China	Neonates with neonatal hyperbilirubinemia	Randomized controlled intervention study	35/35	Neonatal touch combined with early swimming for 1 month	Jaundice burden and bilirubin metabolism	Jaundice index and serum indirect bilirubin after 1 month.
Zhang et al. 2025 (14)	China	Neonates with delayed neurological development	Randomized controlled intervention study	54/54	Swimming combined with early touch therapy for 3 months	Neurodevelopmental and motor function; Growth and anthropometry	NBNA primitive-reflex, general-response, behavioural-ability, active-muscle-tone, and passive-muscle-tone scores; Bayley Mental Development Index; and head circumference, body length, and weight after 3 months.
Pan et al. 2025 (21)	China	Term neonates with hypoxic-ischemic encephalopathy	Randomized controlled intervention study	46/46	Kangaroo nursing plus hydrotherapy and touch until discharge	Growth and anthropometry	Body weight (kg) and height (cm) after the intervention.
Guo 2025 (15)	China	Neonates with jaundice, age <=28 days	Randomized controlled intervention study	30/30	Touch combined with early swimming	Neurodevelopmental and motor function; Jaundice burden and bilirubin metabolism	WS/T 580–2017 developmental-behaviour assessment (adaptability, social behaviour, fine motor, language, gross motor, and total developmental quotient); JH2 percutaneous bilirubin after intervention; and jaundice regression time.
Chen 2025 (22)	China	Neonates with jaundice, age <=28 days	Randomized controlled intervention study	39/39	Early swimming and touch nursing for 5 days	Growth and anthropometry; Jaundice burden and bilirubin metabolism	Jaundice regression time; direct, indirect, and total bilirubin after intervention; jaundice index after intervention; and body weight after intervention.
Yu et al. 2024 (31)	China	Term neonates with bilirubin encephalopathy	Randomized controlled intervention study	46/46	Swimming intervention added to routine rehabilitation care	Jaundice burden and bilirubin metabolism	Total, indirect, and direct bilirubin concentrations after 7 days.
Cao 2016 (29)	China	Preterm infants	Randomized controlled intervention study	43/43	Swimming combined with touch after umbilical cord shedding	Beneficial developmental classification	Gesell developmental classification based on DQ > 86 at 6 months for gross motor, fine motor, adaptive, and language domains.
Wang et al. 2013 (23)	China	Healthy term newborns	Randomized controlled intervention study	144/ 144	Modified touch exercises after swimming	Growth and anthropometry; Jaundice burden and bilirubin metabolism; Feeding, stool transition, and digestion	Head circumference, body length, and weight at 1 month; time for meconium to turn yellow; and day-5 percutaneous bilirubin.
Huang 2012 (16)	China	Healthy term newborns	Randomized controlled intervention study	50/50	Early touch combined with swimming	Neurodevelopmental and motor function; Growth and anthropometry	Gesell gross-motor, fine-motor, cognitive, language, and personal-social scores at 6 months; body length, weight, and head circumference at 6 months.
Hou and Zhang 2010 (32)	China	Normal term newborns	Randomized controlled intervention study	522/ 475	Neonatal swimming once daily for 15–20 min	Jaundice burden and bilirubin metabolism	Day-5 transcutaneous jaundice index measured at the forehead.
Liu 2009 (24)	China	Cesarean-born newborns	Randomized controlled intervention study	100/ 100	Swimming and touch beginning after birth	Growth and anthropometry; Jaundice burden and bilirubin metabolism; Feeding, stool transition, and digestion	Weight and milk intake at 7 days; time to first defecation; time for meconium to turn yellow; and NJ33 transcutaneous jaundice index.
Huang et al. 2006 (25)	China	Newborns with physiological weight loss	Randomized controlled intervention study	102/ 84	Daily hydrotherapy for 15–20 min combined with touch	Growth and anthropometry	Time to recovery from physiological neonatal weight loss (days).
Wen et al. 2005 (26)	China	Normal newborns during hospitalization	Randomized controlled intervention study	100/ 100	Neonatal swimming with swimming exercises plus touch	Growth and anthropometry; Feeding, stool transition, and digestion	Discharge weight; time to first meconium; and time for meconium to turn yellow.
Zhong and Liang 2005 (27)	China	Neonates with moderate or severe scleredema	Randomized controlled intervention study	32/28	Constant-temperature warm-water bath rewarming	Physiological regulation and safety	Temperature-recovery time and scleredema-resolution time, recorded separately for moderate and severe scleredema.
Li 2025 (17)	China	Neonates with neurological developmental delay	Randomized controlled intervention study	40/40	Swimming and early touch for neonatal developmental delay	Neurodevelopmental and motor function	NBNA score after nursing; Mental Development Index and Psychomotor Development Index after 8 weeks.
Yan et al. 2023 (33)	China	Neonates with jaundice	Randomized controlled intervention study	41/41	Touch combined with early swimming for 1 week	Jaundice burden and bilirubin metabolism; Feeding, stool transition, and digestion	Time to first defecation, first yellow stool, and jaundice duration; HP-HD800 transcutaneous jaundice index at intervention end; and colorimetric total and direct bilirubin at intervention end.
Dias et al. 2013 (51)	Brazil	Healthy full-term infants aged 7 to 9 months	Matched controlled pilot intervention study	6/6	Sixteen weekly 45-minute infant swimming lessons over 4 months	Not included in final meta-analysis	Alberta Infant Motor Scale (AIMS) total score and percentile rank before and after four months; not included in the final meta-analysis.
Wu et al. 2020 (52)	China	Healthy full-term newborns	Randomized controlled intervention study	52/52	Early touch plus daily swimming training	Not included in final meta-analysis	Physical-growth indices and neurocognitive-development assessment as reported by the study; controlled data were not sufficiently extractable for the final meta-analysis.
Piao et al. 2005 (53)	China	Normal newborns and 2-month infants	Randomized controlled intervention study	30/30	Infant swimming	Not included in final meta-analysis	Growth and developmental observations in normal newborns and 2-month-old infants; controlled data were not sufficiently extractable for the final meta-analysis.

The aquatic interventions also varied across studies but shared a common feature of planned water-based exposure during infancy or toddlerhood. Interventions included baby or neonatal swimming, swimming exercises, aquatic therapy, family-centered KITE aquatic environment sessions, bucket hydrokinesiotherapy, hydrotherapy and constant-temperature warm-water bathing. In several studies, water-based exposure was combined with touch or massage, kangaroo nursing, rehabilitation, routine neonatal care or home-based early intervention. Therefore, many of the included interventions should be interpreted as formal aquatic activity packages rather than isolated aquatic exposure.

Across the six pre-specified outcome domains, the included studies contributed data to neurodevelopmental and motor function, growth and anthropometry, jaundice burden and bilirubin metabolism, feeding, stool transition and digestion, physiological regulation and safety, and beneficial developmental classification. Several studies contributed outcomes to more than one domain, particularly those that assessed both growth-related and neonatal clinical outcomes.

### Risk of bias

The risk-of-bias assessments are presented in Figures 2A, B. As shown in the domain-level summary graph (Figure 2A), unclear risk of bias was common across several methodological domains, particularly random sequence generation, allocation concealment, incomplete outcome data, selective reporting and other bias. Blinding of participants and personnel was judged as high risk in all included studies, reflecting the practical difficulty of masking caregivers, participants or intervention providers in aquatic-activity interventions. Blinding of outcome assessment showed a mixed pattern, with some studies judged as low risk and most others rated as unclear risk because reporting was insufficient.

**Figure 2:**
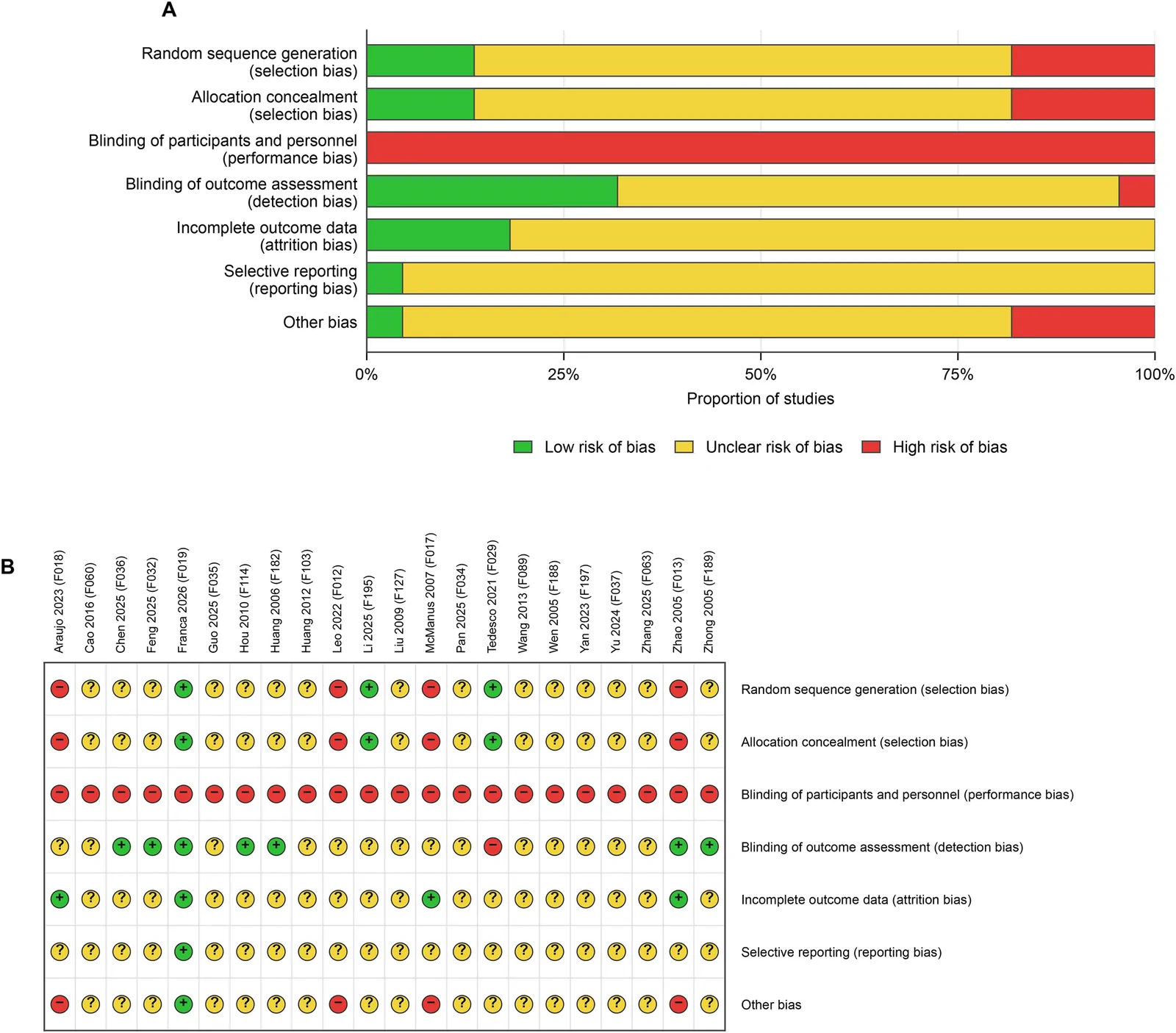
A. Risk-of-bias graph across included controlled intervention studies. B. Study-level traffic-light plot for risk-of-bias judgements.

The study-level traffic-light plot (Figure 2B) further indicated that few studies were judged as low risk across all domains. Several studies had high-risk judgements for random sequence generation, allocation concealment or other bias, while most studies had at least one unclear-risk domain. Overall, the risk-of-bias profile suggests that the included evidence should be interpreted as controlled intervention evidence with important reporting and blinding limitations, rather than as uniformly low-risk causal evidence.

### Health and developmental outcomes and effect-size distribution of aquatic activities

Across the included studies, a total of 87 outcome-level effect sizes were extracted. Each effect size was assigned to the domain of its corresponding outcome according to the pre-specified ICF-CY-informed coding process; detailed coding procedures and domain-assignment criteria are provided in Supplementary Material 1. The most frequently represented domain was neurodevelopmental and motor function, which contributed 29 effect sizes (29/87; 33.3%). Growth and anthropometry and jaundice burden and bilirubin metabolism each contributed 19 effect sizes (19/87; 21.8%). Smaller numbers of effect sizes were available for feeding, stool transition and digestion (8/87; 9.2%), beneficial developmental classification (7/87; 8.0%), and physiological regulation and safety (5/87; 5.7%). The detailed effect-size distribution across domains is presented in Figure 3.

**Figure 3:**
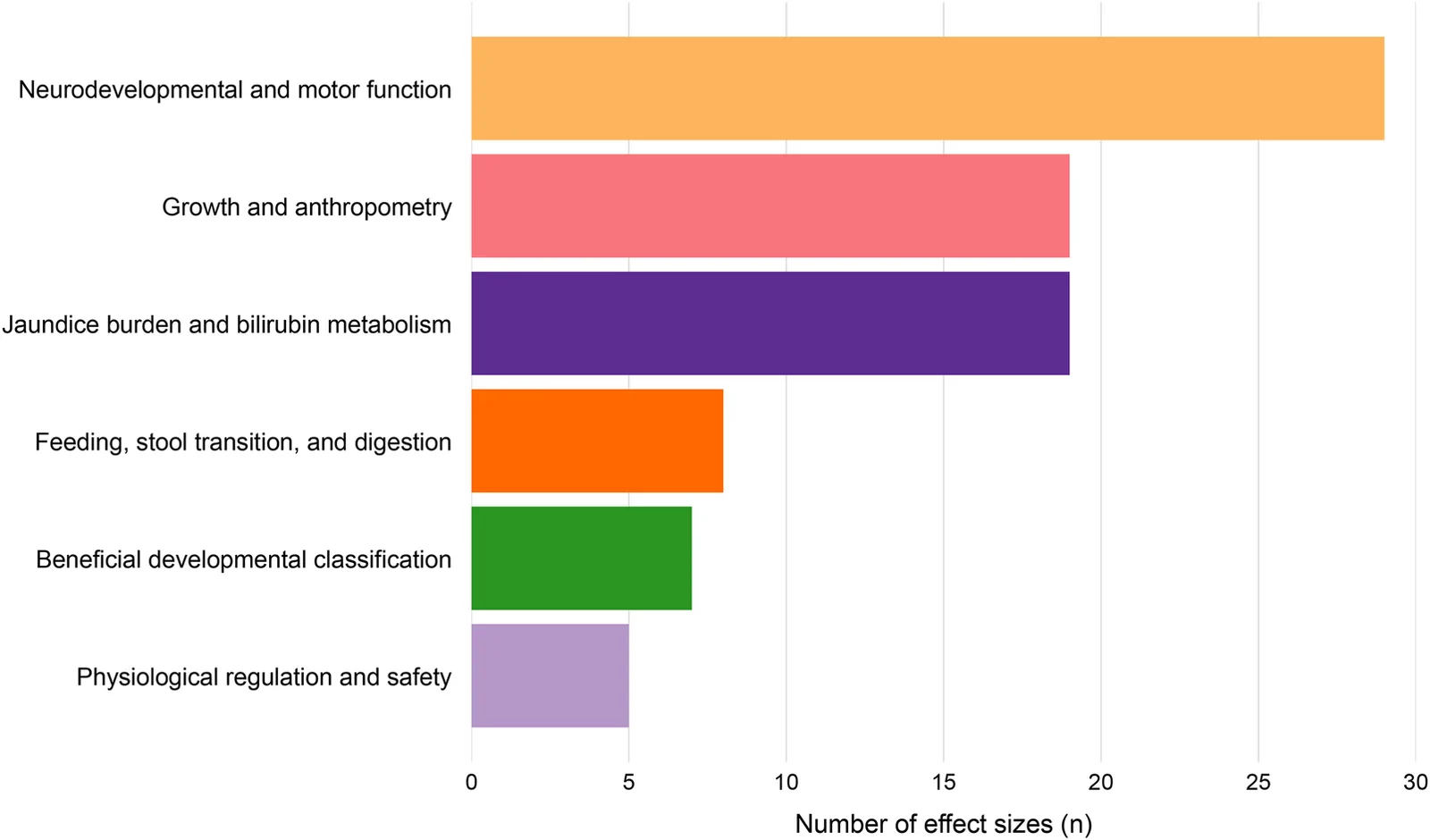
Frequency distribution of extracted effect sizes across the six ICF-CY outcome domains.

### Meta-analyses of aquatic activities across outcome domains

Meta-analyses were conducted to estimate the pooled effects of aquatic activities across the health and developmental outcomes included in this review. The results of these meta-analyses are summarized in Table 2, and the corresponding forest plots are provided in the Supplementary Material 2. The outcome domains analyzed were neurodevelopmental and motor function, growth and anthropometry, physiological regulation and safety, beneficial developmental classification, jaundice burden and bilirubin metabolism, and feeding, stool transition, and digestion.

**Table 2:** A summary of meta-analyses results on each outcome.

Outcomes	Studies (N)	Effect sizes (n)	Estimate (95% CI)	*P* value	I^2^ (%)
Neurodevelopmental and motor function	6	29	0.96 [0.78 to 1.15]	*P* < 0.0001	47.4%
Growth and anthropometry	11	19	0.54 [0.44 to 0.63]	*P* < 0.0001	23.5%
Physiological regulation and safety	2	5	0.85 [0.49 to 1.22]	*P* < 0.0001	9.6%
Beneficial developmental classification	2	7	1.45 [1.29 to 1.62]	*P* < 0.0001	0%
Jaundice burden and bilirubin metabolism	8	19	2.31 [1.37 to 3.26]	*P* < 0.0001	96.2%
Feeding, stool transition, and digestion	4	8	0.81 [0.61 to 1.01]	*P* < 0.0001	48.5%

These meta-analytic findings provide an overview of the magnitude, precision, and consistency of the effects of aquatic activities across different health and developmental domains in infants and toddlers. The number of contributing studies and extracted effect sizes was reported for each domain to make the contribution of multiple outcomes within individual studies transparent.

#### Neurodevelopmental and motor function

In a random-effects meta-analysis of 29 effect sizes from 6 studies, aquatic activities showed a significant favourable effect on neurodevelopmental and motor function in infants and toddlers (SMD = 0.96, 95% CI = 0.78–1.15, *P* < 0.0001; Supplementary Figure S1 in Supplementary Materials 2). This finding suggests that aquatic-activity interventions were associated with better neurodevelopmental and motor outcomes than comparator conditions. The six contributing studies were Leo et al., McManus and Kotelchuck, Zhang et al., Guo, Huang, and Li (12–17). Neurodevelopmental and motor outcomes were assessed using the Peabody Developmental Motor Scales, Second Edition (PDMS-2), the Gross Motor Subscale of the Mullen Scales of Early Learning, the Neonatal Behavioral Neurological Assessment (NBNA), mental and psychomotor developmental indices, and multidomain developmental-behaviour assessments.

Heterogeneity was moderate across the included effect sizes (*I^2^* = 47.4%; *τ^2^* = 0.0781; *Q* = 58.11, *df* = 28, *P* = 0.0007), and leave-one-out sensitivity analysis was therefore not performed. The exploratory funnel plot showed some dispersion in the distribution of effect sizes (Supplementary Figure S2 in Supplementary Materials 2). Overall, the pooled result was statistically robust and consistently favoured aquatic activities, supporting a positive association between formal aquatic activity participation and early neurodevelopmental and motor performance.

#### Growth and anthropometry

In a random-effects meta-analysis of 19 effect sizes from 11 studies, aquatic activities showed a significant favourable effect on growth and anthropometry outcomes in infants and toddlers (SMD = 0.54, 95% CI = 0.44–0.63, *P* < 0.0001; refer to Supplementary Figure S3 in Supplementary Material 2). This result indicates that aquatic-activity interventions were associated with more favourable growth-related and anthropometric outcomes than comparator conditions. The 11 contributing studies were Zhao et al., França et al., Tedesco et al., Zhang et al., Pan et al., Chen, Wang et al., Huang (2012), Liu, Huang et al. (2006), and Wen et al. (14, 16, 18–26). Growth was measured using body weight or weight gain, body length or height, head circumference, and time to recovery from physiological neonatal weight loss, based on infant scales, routine anthropometric assessment, and recorded recovery time at the study-specific follow-up points.

Heterogeneity was low to moderate and remained below the prespecified threshold for leave-one-out sensitivity analysis (*I*^2^ = 23.5%; *τ*^2^ = 0.0128; *Q* = 29.34, *df* = 18, *P* = 0.0444); therefore, leave-one-out sensitivity analysis was not performed for this domain. The exploratory funnel plot showed a generally concentrated distribution of effect sizes around the pooled estimate, with some dispersion across studies (refer to Supplementary Figure S4 in Supplementary Material 2). Overall, the pooled estimate consistently favoured aquatic activities, supporting a positive effect of formal aquatic activity participation on early growth and anthropometric development.

#### Physiological regulation and safety

In the physiological regulation and safety domain, the random-effects meta-analysis included 5 effect sizes from 2 studies. Aquatic activities demonstrated a significant favourable effect on physiological regulation and safety-related outcomes in infants and toddlers (SMD = 0.85, 95% CI = 0.49–1.22, *P* < 0.0001; refer to Supplementary Figure S5 in Supplementary Material 2). This result suggests that formal aquatic activity interventions may contribute to more favourable physiological regulation and safety-related clinical indicators compared with comparator conditions. The two contributing studies were Tedesco et al. and Zhong and Liang (20, 27). Outcomes comprised oxygen saturation measured by pulse oximetry in Tedesco et al. and temperature-recovery and scleredema-resolution times recorded separately for moderate and severe neonatal scleredema in Zhong and Liang.

The heterogeneity estimate was low and did not reach the prespecified threshold for leave-one-out sensitivity analysis (*I^2^* = 9.6%; *τ^2^* = 0.0156; *Q* = 4.31, *df* = 4, *P* = 0.3652); therefore, no leave-one-out sensitivity analysis was conducted for this domain. The exploratory funnel plot showed a relatively compact distribution of effect sizes around the pooled estimate, with limited dispersion across studies (refer to Supplementary Figure S6 in Supplementary Material 2). Overall, the pooled effect consistently supported the beneficial role of aquatic activities in physiological regulation and safety-related outcomes.

#### Beneficial developmental classification

For beneficial developmental classification, the random-effects meta-analysis synthesized 7 effect sizes from 2 studies. Aquatic activities were associated with a significantly higher likelihood of favourable developmental classification among infants and toddlers (RR = 1.45, 95% CI = 1.29–1.62, *P* < 0.0001; refer to Supplementary Figure S7 in Supplementary Material 2). This result indicates that infants and toddlers receiving aquatic-activity interventions were more likely to be classified as developmentally favourable than those in comparator groups. The two contributing studies were Araujo et al. and Cao (28, 29). Araujo et al. classified typical development using overall neuropsychomotor development (NPMD), the Alberta Infant Motor Scale (AIMS), and the Denver II Developmental Screening Test, whereas Cao used Gesell developmental quotients and the prespecified threshold of DQ > 86 across developmental domains.

No meaningful heterogeneity was observed in this domain (*I^2^* = 0.0%; *τ^2^* = 0.0000; *Q* = 4.18, *df* = 6, *P* = 0.6528). The exploratory funnel plot showed that most effect sizes were distributed close to the pooled estimate, with limited between-effect variation (refer to Supplementary Figure S8 in Supplementary Material 2). Overall, the pooled result showed a clear and consistent advantage of aquatic activities for beneficial developmental classification.

#### Jaundice burden and bilirubin metabolism

For jaundice burden and bilirubin metabolism, 19 effect sizes from 8 studies were synthesized using a random-effects model. After direction alignment of jaundice- and bilirubin-related indicators, aquatic activities showed a significant favourable effect in this domain (SMD = 2.31, 95% CI = 1.37–3.26, *P* < 0.0001; refer to Supplementary Figure S9 in Supplementary Material 2). This finding indicates that formal aquatic activity interventions were associated with more favourable jaundice-related clinical indicators and bilirubin metabolism than comparator conditions. The eight contributing studies were Feng, Guo, Chen, Yu et al., Wang et al., Hou and Zhang, Liu, and Yan et al. (15, 22–24, 30–33). Jaundice-related outcomes were measured using transcutaneous bilirubin or jaundice indices, laboratory total, direct, or indirect bilirubin concentrations, and time-based measures such as jaundice duration or regression time.

Substantial heterogeneity was observed across the included effect sizes (*I^2^* = 96.2%; *τ^2^* = 2.3655; *Q* = 655.10, *df* = 18, *P* < 0.0001). Leave-one-out sensitivity analysis was therefore performed (refer to Supplementary Table S1 in Supplementary Material 3). After omitting each study in turn, the pooled estimates remained statistically significant and consistently favoured aquatic activities, ranging from SMD = 2.01 to 2.62. The corresponding *I^2^* values remained high, ranging from 95.7% to 96.6%, suggesting that no single study fully accounted for the observed heterogeneity.

As shown in Supplementary Table S2 of Supplementary Material 3, subgroup analyses further showed favourable pooled effects across outcome measurement types, population characteristics and intervention packages. The pooled effect was significant for both bilirubin or jaundice index levels (SMD = 2.33, 95% CI = 1.32–3.33, *P* < 0.0001) and jaundice duration or regression time (SMD = 1.90, 95% CI = 1.19–2.61, *P* < 0.0001). Favourable effects were also observed in healthy or non-clinical newborns (SMD = 1.67, 95% CI = 0.01–3.33, *P* = 0.048) and in jaundice or hyperbilirubinemia clinical samples (SMD = 2.58, 95% CI = 1.41–3.76, *P* < 0.0001). By intervention type, both aquatic exposure combined with touch, rehabilitation or bundled care (SMD = 1.86, 95% CI = 0.98–2.75, *P* < 0.0001) and aquatic exposure without added touch (SMD = 3.76, 95% CI = 2.94–4.58, *P* < 0.0001) showed significant favourable effects. The exploratory funnel plot showed a dispersed distribution of effect sizes, consistent with the substantial variability observed in the forest plot (refer to Supplementary Figure S10 in Supplementary Material 2). Overall, this domain showed the largest pooled effect among the six outcome domains and consistently supported a beneficial effect of aquatic activities on jaundice burden and bilirubin metabolism.

#### Feeding, stool transition, and digestion

For feeding, stool transition, and digestion, the random-effects meta-analysis incorporated 8 effect sizes from 4 studies. After aligning the direction of outcomes for which lower values indicated more favourable clinical status, aquatic activities showed a significant beneficial effect in this domain (SMD = 0.81, 95% CI = 0.61–1.01, *P* < 0.0001; refer to Supplementary Figure S11 in Supplementary Material 2). This result suggests that formal aquatic activity interventions were associated with more favourable feeding-related, stool-transition, and digestive outcomes compared with comparator conditions. The four contributing studies were Wang et al., Liu, Wen et al., and Yan et al. (23, 24, 26, 33). These studies recorded milk intake and clinically timed gastrointestinal outcomes, including time to first defecation and time for meconium or stool to turn yellow.

The heterogeneity estimate was moderate but remained below the prespecified threshold for leave-one-out sensitivity analysis (*I^2^* = 48.5%; *τ^2^* = 0.0266; *Q* = 11.80, *df* = 7, *P* = 0.1075). The exploratory funnel plot showed that most effect sizes were distributed around the pooled estimate, with some variation across individual effects (refer to Supplementary Figure S12 in Supplementary Material 2). Taken together, the pooled findings supported a favourable effect of aquatic activities on early feeding, stool transition, and digestive regulation.

## Discussion

This ICF-CY-informed systematic review and meta-analysis synthesized controlled evidence on the effects of formal aquatic activities on health and developmental outcomes in infants and toddlers younger than 36 months. Overall, aquatic activities were associated with favourable outcomes across neurodevelopmental, growth, physiological, developmental classification, jaundice-related, and feeding or digestive domains.

### Neurodevelopmental and motor function

Our findings showed a significant favourable effect of formal aquatic activities on neurodevelopmental and motor function in infants and toddlers (4). This finding aligns with previous research indicating that structured aquatic activities may support early motor development, functional mobility, postural control and broader developmental performance (12). The water environment may provide a developmentally supportive context in which infants can move with reduced gravitational constraints, greater freedom of limb movement and continuous sensory input (5). These conditions may be particularly relevant during early life, when motor exploration and sensorimotor experiences are closely linked to emerging neurological and functional development (2).

The pooled neurodevelopmental and motor-function result was based on 29 effect estimates from six studies. Five of the six studies contributed at least one statistically significant estimate in the favourable direction; four contributed exclusively significant estimates, one showed mixed findings, and one contributed a non-significant estimate. Overall, 24 of the 29 effect estimates were statistically significant (12–17).

Early motor acquisition depends heavily on repeated, variable and supported sensorimotor exploration, and aquatic environments alter the biomechanical conditions of infant movement (34). Buoyancy reduces weight bearing demands, whereas water resistance provides continuous, low intensity proprioceptive input during limb and trunk activity (5). Under these conditions, infants may have greater opportunities to initiate, repeat and adapt movement patterns that are less accessible during activity on land, thereby supporting postural control, motor coordination and functional mobility (7). Warm water exposure and handling supported by caregivers or therapists may further enhance engagement and arousal regulation, linking physical practice with sensory and social interaction (19). The moderate heterogeneity observed in this domain may reflect differences in age, baseline developmental status, intervention dose and assessment instruments; nevertheless, the consistently favourable pooled effect supports the use of formal aquatic activities as a complementary approach to promoting early neurodevelopmental and motor outcomes.

### Growth and anthropometry

Formal aquatic activities were associated with favourable growth and anthropometric outcomes in infants and toddlers (35). This finding is consistent with previous research suggesting that structured aquatic activities in early life may support physical growth indicators such as weight, length and head circumference (18). Compared with routine care alone, aquatic activities combine gentle movement, warm water exposure and caregiver supported handling, which may promote behavioural regulation, improve comfort and increase opportunities for low intensity physical activity without imposing excessive mechanical load (36).

The growth and anthropometry synthesis included 19 effect estimates from 11 studies. Nine studies contributed at least one statistically significant favourable estimate: eight contributed exclusively significant estimates, one showed mixed findings, and two contributed only non-significant estimates. Overall, 16 of the 19 effect estimates were statistically significant (14, 16, 18–26).

The relatively low heterogeneity observed in this domain suggests that the positive association was broadly consistent across different growth-related measures and study contexts. Still, growth and anthropometry are influenced by multiple factors, including gestational age, baseline health status, nutritional intake and routine care quality. Therefore, the observed effect should be understood as the potential contribution of aquatic activity within a broader caregiving and developmental environment, rather than as an isolated determinant of growth (37). Future studies should further clarify which intervention dose, timing and population characteristics are most relevant to growth related benefits (35).

### Physiological regulation and safety

The present review identified a significant favourable effect of formal aquatic activities on physiological regulation and safety related outcomes (4). This result suggests that, when delivered in a structured and supervised manner, aquatic activities may be compatible with physiological stability in infants and toddlers and may contribute to selected indicators of regulation. This finding is consistent with previous research indicating that warm water exposure and gentle handling can support autonomic balance, comfort and behavioural state regulation during early life (38).

This domain included five effect estimates from two studies. Both studies contributed at least one statistically significant favourable estimate; one study contributed exclusively significant estimates, whereas the other showed mixed findings. Four of the five effect estimates were statistically significant (20, 27).

The mechanisms underlying this effect are likely related to the combined influence of thermal, sensory and postural conditions in the aquatic environment (5). Warm water may reduce stress responses and facilitate relaxation, while continuous tactile and proprioceptive input may help stabilize arousal and support smoother transitions between activity and rest (39). The low heterogeneity observed in this domain suggests that the pooled effect was relatively consistent across the included studies. However, because safety outcomes are highly dependent on supervision quality, water temperature, session duration and infant health status, these findings should be interpreted within the context of structured and professionally guided aquatic activities rather than unsupervised water exposure (40).

### Beneficial developmental classification

The present synthesis further indicated that formal aquatic activities were associated with a higher likelihood of beneficial developmental classification in infants and toddlers. This pattern is consistent with the positive effect observed for neurodevelopmental and motor function, suggesting that aquatic activities may not only improve continuous developmental scores but also increase the probability of reaching favourable developmental categories (12). Such classification outcomes are clinically meaningful because they reflect whether infants or toddlers meet expected developmental criteria rather than only the magnitude of change on a scale (41).

Beneficial developmental classification was evaluated using seven effect estimates from two studies. Both studies consistently favoured the aquatic intervention, and all seven estimates were statistically significant (28, 29).

This association may reflect the cumulative influence of repeated movement practice, sensory stimulation and caregiver supported interaction within aquatic sessions (42). By creating opportunities for infants to practise postural control, limb movement and functional responses in a supportive environment, aquatic activities may help some children move beyond developmental thresholds used in screening or classification systems (7). At the same time, this domain was represented by a small number of studies, and classification results can be influenced by the assessment instrument, cut off values and timing of evaluation (43). Future studies should therefore use standardized developmental classification criteria and longer follow up to determine whether these favourable classifications are sustained over time.

### Jaundice burden and bilirubin metabolism

In this review, jaundice burden and bilirubin metabolism showed the largest pooled effect among the six outcome domains, with results favouring formal aquatic activities after direction alignment of jaundice related indicators. This finding is consistent with previous research suggesting that structured warm water bathing or neonatal swimming may help reduce bilirubin related burden in newborns (44). The effect remained favourable in leave one out sensitivity analyses, and subgroup analyses also showed significant pooled effects across different outcome measurement types, population characteristics and intervention packages, indicating that the overall direction of association was not driven by a single study or subgroup (45).

The jaundice and bilirubin synthesis included 19 effect estimates from eight studies. All eight studies contributed statistically significant favourable findings, and all 19 effect estimates excluded the null value. Nevertheless, the substantial heterogeneity indicates that the magnitude, rather than the direction, of the association varied considerably across studies (15, 22–24, 30–33).

The observed benefit may be partly related to improved behavioural regulation, increased comfort and enhanced gastrointestinal activity during or after aquatic exposure (33). Gentle movement and warm water stimulation may support feeding tolerance, bowel activity and stool transition, which are clinically relevant because bilirubin elimination is closely linked to intestinal excretion in early life (46). At the same time, this domain showed substantial heterogeneity, suggesting that the magnitude of effect varied considerably across studies. Differences in baseline jaundice severity, timing of intervention, concurrent phototherapy or routine care, and the specific bilirubin or jaundice measures used may explain part of this variability (47). Therefore, aquatic activities may have potential as a supportive care component for jaundice related outcomes, but future studies should clarify the optimal timing, frequency and clinical context of implementation. Differences in assessment methods may also have contributed to this variability because bilirubin concentrations, jaundice indices, and time-to-resolution measures capture related but not identical aspects of jaundice burden. Although subgroup analyses by outcome measurement type continued to show favourable pooled effects, substantial heterogeneity remained, suggesting that measurement method alone did not account for the observed variability.

### Feeding, stool transition, and digestion

Finally, this review found that formal aquatic activities were associated with favourable feeding, stool transition and digestion outcomes. This finding is consistent with previous research suggesting that warm water exposure, gentle movement and supportive handling may contribute to gastrointestinal comfort and early feeding adaptation in infants (48). These outcomes are particularly relevant in the neonatal period, when feeding tolerance, bowel activity and stool transition are closely connected with physiological adaptation after birth (49).

Feeding, stool transition, and digestion were represented by eight effect estimates from four studies. All four studies contributed statistically significant favourable findings, and all eight effect estimates excluded the null value (23, 24, 26, 33).

The observed association may be related to the combined regulatory effects of warmth, movement and tactile stimulation (36). Warm water may promote relaxation and reduce discomfort, while gentle aquatic movement may support abdominal activity and intestinal motility (24). Supportive handling by caregivers or nurses during aquatic sessions may also improve behavioural state regulation, which can indirectly facilitate feeding and digestive function (50). The moderate heterogeneity in this domain suggests some variation across studies, possibly related to differences in infant health status, intervention timing, feeding practices and outcome definitions (23). Overall, the pooled effect supports the potential value of formal aquatic activities as a supportive strategy for early feeding and digestive regulation, although more standardized outcome measurement is needed in future studies.

### Strengths

The main strength of this study is that it is, to our knowledge, the first systematic review and meta-analysis to comprehensively examine the effects of formal aquatic activities on health and developmental outcomes in infants and toddlers younger than 36 months. By synthesizing controlled evidence across neurodevelopmental, growth, physiological, jaundice-related, feeding and developmental classification outcomes, this study addresses an important gap in the existing literature, where aquatic activities in early life have often been evaluated within separate clinical or developmental contexts. The resulting framework provides a structured basis for interpreting the potential role of aquatic activities in early health and development. Moreover, the use of the ICF-CY framework allowed heterogeneous outcomes to be organized into clinically meaningful domains, improving comparability across studies with different populations, interventions and measurement tools. The inclusion of both English and Chinese literature, together with direction aligned effect sizes and additional sensitivity and subgroup analyses for the most heterogeneous domain, further strengthened the comprehensiveness and interpretability of the review.

### Limitations

However, several limitations should be acknowledged. The included studies varied in participant characteristics, intervention components and outcome measures, which may have contributed to differences in effect estimates across domains. In some studies, aquatic exposure was delivered together with other care components, making it difficult to determine the separate contribution of the aquatic activity itself. In addition, methodological reporting was not always detailed, and some outcome domains were supported by a limited number of studies. Although sensitivity and subgroup analyses helped examine the most heterogeneous domain, future studies with more standardized intervention protocols, outcome measures and longer follow-up are still needed to strengthen the evidence base. In addition, applying RoB 1 uniformly across randomized and non-randomized controlled studies may not capture all design-specific sources of bias in the non-randomized evidence.

Some variation in assessment methods should be acknowledged when interpreting these domain-level findings. Although outcomes were grouped according to their ICF-CY classification and broader clinical relevance, measures within the same domain may not be directly comparable. Standardization using SMDs enabled the quantitative synthesis of outcomes assessed using different measures, although some heterogeneity in their clinical interpretation may remain.

## Conclusion

This ICF-CY-informed systematic review and meta-analysis provides a comprehensive synthesis of controlled evidence on formal aquatic activities in infants and toddlers younger than 36 months. The findings suggest that aquatic activities may have favourable effects across multiple health and developmental domains, including neurodevelopmental and motor function, growth and anthropometry, physiological regulation and safety, beneficial developmental classification, jaundice burden and bilirubin metabolism, and feeding, stool transition and digestion. By organizing heterogeneous outcomes into clinically meaningful domains, this review offers a clearer understanding of the potential role of aquatic activities in early life care and development. Future studies should adopt more rigorous designs, standardized intervention protocols and longer follow-up to confirm these effects and clarify the populations and clinical contexts in which aquatic activities may be most beneficial.
