# Supplementary material for: Effects of formal aquatic activities on health and developmental outcomes in infants and toddlers younger than 36 months: a systematic review and meta-analysis
Source: Front Pediatr. 2026 Sep 30;14:1910257. doi: 10.3389/fped.2026.1910257 (PMC13662420; doi:10.3389/fped.2026.1910257)
Supplement: Supplementary file 1 [file Supplementaryfile1.docx]

**Supplementary Materials 1**

***Search Strategy (May 2026)***

***PubMed <December 2022 to May 25, 2026>→1543***

("Infant"[Mesh] OR "Child, Preschool"[Mesh] OR "Infant, Newborn"[Mesh] OR "Infant, Premature"[Mesh] OR infant*[tiab] OR baby[tiab] OR babies[tiab] OR toddler*[tiab] OR newborn*[tiab] OR neonatal[tiab] OR neonate*[tiab] OR preterm[tiab] OR premature[tiab] OR "early childhood"[tiab] OR "young child*"[tiab] OR pediatric[tiab] OR paediatric[tiab]) AND ("Swimming"[Mesh] OR "Hydrotherapy"[Mesh] OR "Baths"[Mesh] OR "Immersion"[Mesh] OR "Swimming Pools"[Mesh] OR swim*[tiab] OR aquatic*[tiab] OR hydrotherap*[tiab] OR hydro-therap*[tiab] OR "water therap*"[tiab] OR "water exercise*"[tiab] OR "water activit*"[tiab] OR "water intervention*"[tiab] OR "pool activit*"[tiab] OR "pool exercise*"[tiab] OR "baby swim*"[tiab] OR "infant swim*"[tiab] OR "toddler swim*"[tiab] OR "aquatic exercise*"[tiab] OR "aquatic activit*"[tiab] OR "aquatic therap*"[tiab] OR "aquatic intervention*"[tiab] OR "aquatic program*"[tiab] OR "aquatic programme*"[tiab] OR "aquatic stimulation"[tiab] OR "aquatic environment*"[tiab] OR "water immersion"[tiab] OR "water submersion*"[tiab] OR bath[tiab] OR baths[tiab] OR bathing[tiab] OR "bucket hydrotherap*"[tiab] OR "bucket bath*"[tiab] OR "hammock bath*"[tiab] OR "swimming lesson*"[tiab] OR "swimming program*"[tiab] OR "swimming programme*"[tiab] OR "swimming class*"[tiab] OR "swimming course*"[tiab] OR "in water"[tiab] OR "water-based"[tiab] OR "water based"[tiab] OR spa[tiab]) NOT (animals[mesh] NOT humans[mesh])

***Ovid MEDLINE(R) ALL <December 2022 to May 25, 2026>→1311***

1. exp Infant/ or exp Child, Preschool/ or exp Infant, Newborn/ or exp Infant, Premature/
2. (infant* or baby or babies or toddler* or newborn* or neonatal or neonate* or preterm or premature or early childhood or young child* or pediatric or paediatric).ti,ab,kf.
3. 1 or 2
4. exp Swimming/ or exp Hydrotherapy/ or exp Baths/ or exp Immersion/
5. (swim* or aquatic* or hydrotherap* or hydro-therap* or water therap* or water exercise* or water activit* or water intervention* or pool activit* or pool exercise* or pool therap* or baby swim* or infant swim* or toddler swim* or aquatic exercise* or aquatic activit* or aquatic therap* or aquatic intervention* or aquatic program* or aquatic programme* or aquatic stimulation or aquatic environment* or water immersion or water submersion* or bath or baths or bathing or bucket hydrotherap* or bucket bath* or hammock bath* or swimming lesson* or swimming program* or swimming programme* or swimming class* or swimming course* or in water or water-based or water based or spa).ti,ab,kf.
6. 4 or 5
7. 3 and 6
8. exp Animals/ not (exp Animals/ and exp Humans/)
9. 7 not 8

***Ovid EMBASE <December 2022 to May 25, 2026>→2100***

1. exp Infant/ or exp Child, Preschool/ or exp Infant, Newborn/ or exp Infant, Premature/
2. (infant* or baby or babies or toddler* or newborn* or neonatal or neonate* or preterm or premature or early childhood or young child* or pediatric or paediatric).ti,ab,kf.
3. 1 or 2
4. exp Swimming/ or exp Hydrotherapy/ or exp Baths/ or exp Immersion/
5. (swim* or aquatic* or hydrotherap* or hydro-therap* or water therap* or water exercise* or water activit* or water intervention* or pool activit* or pool exercise* or pool therap* or baby swim* or infant swim* or toddler swim* or aquatic exercise* or aquatic activit* or aquatic therap* or aquatic intervention* or aquatic program* or aquatic programme* or aquatic stimulation or aquatic environment* or water immersion or water submersion* or bath or baths or bathing or bucket hydrotherap* or bucket bath* or hammock bath* or swimming lesson* or swimming program* or swimming programme* or swimming class* or swimming course* or in water or water-based or water based or spa).ti,ab,kf.
6. 4 or 5
7. 3 and 6
8. exp Animals/ not (exp Animals/ and exp Humans/)
9. 7 not 8

***PsycINFO (via ProQuest) <December 2022 to May 25, 2026>→123***

MAINSUBJECT.EXACT("Infant Development") OR MAINSUBJECT.EXACT("Neonatal Development") OR MAINSUBJECT.EXACT("Early Childhood Development") OR ti(infant* OR baby OR babies OR toddler* OR newborn* OR neonatal OR neonate*) OR ti(preterm OR premature OR "early childhood" OR "young child*" OR pediatric) OR ab(infant* OR baby OR babies OR toddler* OR newborn* OR neonatal OR neonate*) OR ab(preterm OR premature OR "early childhood" OR "young child*" OR pediatric))

AND

(MAINSUBJECT.EXACT("Swimming") OR MAINSUBJECT.EXACT("Hydrotherapy") OR ti(swim* OR aquatic* OR hydrotherap* OR hydro-therap* OR "water therap*") OR ti("water exercise*" OR "water activit*" OR "baby swim*" OR "infant swim*") OR ti("aquatic exercise*" OR "aquatic activit*" OR "aquatic therap*") OR ti("aquatic intervention*" OR "aquatic program*" OR "water immersion") OR ti(bath OR baths OR bathing OR "bucket hydrotherap*" OR "hammock bath*") OR ti("swimming lesson*" OR "swimming program*" OR "swimming class*") OR ti("in water" OR "water-based" OR spa) OR ab(swim* OR aquatic* OR hydrotherap* OR hydro-therap* OR "water therap*") OR ab("water exercise*" OR "water activit*" OR "baby swim*" OR "infant swim*") OR ab("aquatic exercise*" OR "aquatic activit*" OR "aquatic therap*") OR ab("aquatic intervention*" OR "aquatic program*" OR "water immersion") OR ab(bath OR baths OR bathing OR "bucket hydrotherap*" OR "hammock bath*") OR ab("swimming lesson*" OR "swimming program*" OR "swimming class*") OR ab("in water" OR "water-based" OR spa))

NOT

(MAINSUBJECT.EXACT("Animal Ethology") OR MAINSUBJECT.EXACT("Animal Behavior"))

NOT ti,ab(rat OR rats OR mouse OR mice OR rodent* OR animal*)

***Scopus <December 2022 to May 25, 2026>→2013***

TITLE-ABS-KEY(

( infant* OR baby OR babies OR toddler* OR newborn* OR neonatal OR neonate* OR preterm OR premature OR "early childhood" OR "young child*" OR pediatric OR paediatric )

AND

( swim* OR aquatic* OR hydrotherap* OR hydro-therap* OR "water therap*" OR "water exercise*" OR "water activit*" OR "water intervention*" OR "pool activit*" OR "pool exercise*" OR "pool therap*" OR "baby swim*" OR "infant swim*" OR "toddler swim*" OR "aquatic exercise*" OR "aquatic activit*" OR "aquatic therap*" OR "aquatic intervention*" OR "aquatic program*" OR "aquatic programme*" OR "aquatic stimulation" OR "aquatic environment*" OR "water immersion" OR "water submersion*" OR bath OR baths OR bathing OR "bucket hydrotherap*" OR "bucket bath*" OR "hammock bath*" OR "swimming lesson*" OR "swimming program*" OR "swimming programme*" OR "swimming class*" OR "swimming course*" OR "in water" OR "water-based" OR "water based" OR spa ))

AND NOT

INDEXTERMS( animal* )

***SportDiscus (via EBSCOhost) <December 2022 to May 25, 2026>→230***

( DE "INFANTS" OR DE "NEWBORN infants" OR DE "PREMATURE infants" OR DE "TODDLERS" OR TI,AB( infant* OR baby OR babies OR toddler* OR newborn* OR neonatal OR neonate* OR preterm OR premature OR "early childhood" OR "young child*" OR pediatric OR paediatric ) ) AND ( DE "SWIMMING" OR DE "AQUATIC exercises" OR DE "HYDROTHERAPY" OR DE "WATER immersion" OR TI,AB( swim* OR aquatic* OR hydrotherap* OR hydro-therap* OR "water therap*" OR "water exercise*" OR "water activit*" OR "water intervention*" OR "pool activit*" OR "baby swim*" OR "infant swim*" OR "toddler swim*" OR "aquatic exercise*" OR "aquatic activit*" OR "aquatic therap*" OR "aquatic intervention*" OR "aquatic program*" OR "aquatic stimulation" OR "aquatic environment*" OR "water immersion" OR "water submersion*" OR bath OR baths OR bathing OR "bucket hydrotherap*" OR "bucket bath*" OR "hammock bath*" OR "swimming lesson*" OR "swimming program*" OR "swimming class*" OR "swimming course*" OR "in water" OR "water-based" OR spa ) ) AND ( TI,AB( human* OR patient* OR participant* OR subject* OR person* OR people OR "clinical trial" OR "cohort study" OR "case-control" OR "randomized controlled trial" ) )

***Web of Science Core Collection <December 2022 to May 25, 2026>→1258***

TS=(( infant* OR baby OR babies OR toddler* OR newborn* OR neonatal OR neonate* OR preterm OR premature OR "early childhood" OR "young child*" OR pediatric OR paediatric )

AND

( swim* OR aquatic* OR hydrotherap* OR hydro-therap* OR "water therap*" OR "water exercise*" OR "water activit*" OR "water intervention*" OR "pool activit*" OR "pool exercise*" OR "pool therap*" OR "baby swim*" OR "infant swim*" OR "toddler swim*" OR "aquatic exercise*" OR "aquatic activit*" OR "aquatic therap*" OR "aquatic intervention*" OR "aquatic program*" OR "aquatic programme*" OR "aquatic stimulation" OR "aquatic environment*" OR "water immersion" OR "water submersion*" OR bath OR baths OR bathing OR "bucket hydrotherap*" OR "bucket bath*" OR "hammock bath*" OR "swimming lesson*" OR "swimming program*" OR "swimming programme*" OR "swimming class*" OR "swimming course*" OR "in water" OR "water-based" OR "water based" OR spa ))

NOT

TS=( rat OR rats OR mouse OR mice OR rodent* OR canine* OR equine* OR animal* )

***EBSCOhost <December 2022 to May 25, 2026>→178***

( MH "Infant+" OR MH "Child, Preschool+" OR MH "Infant, Newborn+" OR TI,AB( infant* OR baby OR babies OR toddler* OR newborn* OR neonatal OR neonate* OR preterm OR premature OR "early childhood" OR "young child*" OR pediatric OR paediatric ) ) AND ( MH "Swimming+" OR MH "Hydrotherapy+" OR MH "Bathing and Baths+" OR MH "Immersion+" OR TI,AB( swim* OR aquatic* OR hydrotherap* OR hydro-therap* OR "water therap*" OR "water exercise*" OR "water activit*" OR "water intervention*" OR "pool activit*" OR "baby swim*" OR "infant swim*" OR "toddler swim*" OR "aquatic exercise*" OR "aquatic activit*" OR "aquatic therap*" OR "aquatic intervention*" OR "aquatic program*" OR "aquatic stimulation" OR "aquatic environment*" OR "water immersion" OR "water submersion*" OR bath OR baths OR bathing OR "bucket hydrotherap*" OR "bucket bath*" OR "hammock bath*" OR "swimming lesson*" OR "swimming program*" OR "swimming class*" OR "swimming course*" OR "in water" OR "water-based" OR spa ) ) AND ( TI,AB( human* OR patient* OR participant* OR subject* OR person* OR people OR "clinical trial" OR "cohort study" OR "case-control" OR "randomized controlled trial" OR "human study" OR "human subjects" ) )

***CNKI 中国知网 (China National Knowledge Infrastructure)<1915 to May 25, 2026>→1499***

SU=(‘婴儿’+‘婴幼儿’+‘幼儿’+‘新生儿’+‘早产儿’+‘婴幼’+‘小儿’+‘学龄前儿童’+‘低龄儿童’+‘幼童’)*(‘游泳’+‘水上活动’+‘水上运动’+‘水中运动’+‘水中活动’+‘水疗’+‘水浴’+‘浸浴’+‘沐浴’+‘洗澡’+‘桶浴’+‘婴儿游泳’+‘婴儿水疗’+‘戏水’+‘水中康复’+‘水中锻炼’+‘水中游戏’+‘水中干预’+‘水上课程’+‘游泳课’+‘游泳训练’+‘泳池活动’+‘温泉’+‘SPA’)

**Supplementary Material: Outcome Domain Coding Process**

**1. Rationale for Using a Theoretical Framework**

The present systematic review addresses the effects of formal aquatic activities on health and developmental outcomes in infants and toddlers younger than 36 months. Primary studies in this field have assessed highly heterogeneous outcomes spanning anthropometric indices (e.g., body weight, length, BMI), biochemical markers (e.g., serum bilirubin, transcutaneous bilirubin), developmental scale scores (e.g., Peabody Developmental Motor Scales, Alberta Infant Motor Scale, Gesell Developmental Schedule), physiological parameters (e.g., heart rate, SpO₂), and behavioural measures (e.g., sleep duration, crying time). Without a structured classification system, outcome grouping risks being arbitrary, which may obscure clinically meaningful patterns and reduce the transparency of evidence synthesis.

To address this challenge, we used the World Health Organization’s International Classification of Functioning, Disability and Health for Children and Youth (ICF-CY; WHO, 2007) to code and categorise health and developmental outcomes extracted from primary studies.

The ICF-CY is the official WHO classification system for describing health and health-related states in children and youth. It organises outcomes into three core dimensions: Body Functions (b codes, chapters b1–b8), Body Structures (s codes, chapters s1–s8), and Activities and Participation (d codes, chapters d1–d9). The ICF-CY provides a biopsychosocial taxonomy that has been extensively applied in paediatric systematic reviews to organise heterogeneous outcome data (e.g., Schiariti et al., 2015; Stucki et al., 2008). Its structure is particularly well suited to the present review because aquatic interventions may influence outcomes at multiple ICF-CY levels—from metabolic functions (b5: bilirubin metabolism) to body structures (s7: growth parameters) to mobility activities (d4: motor skill acquisition).

**2. Three-Step Outcome Coding Process**

The coding process followed three sequential steps.Step 1: Raw outcome subcategories mapped to ICF-CY codes. Two reviewers independently mapped each of the 15 distinct outcome subcategories (defined a priori in the extraction codebook) to its closest ICF-CY chapter and second-level codes. The mapping was guided by the official ICF-CY code definitions (WHO, 2007). Discrepancies were resolved through discussion until consensus was reached.

**Table S1.** Step 1 — Mapping of extracted outcome subcategories to ICF-CY codes.

| **Step** | **Outcome Subcategory**  **(Codebook)** | **ICF Chapter** | **Key ICF Codes (2nd level)** |
| --- | --- | --- | --- |
| **1** | *growth_or_body_composition* | **b5 + s7** | b530 Weight maintenance functions; s7 Structures related to movement |
| **1** | *bilirubin_or_jaundice* | **b5** | b540 General metabolic functions |
| **1** | *gross_motor / fine_motor / aquatic_motor_skill* | **d4 + b7** | d410–d450 Mobility; b710–b770 Neuromusculoskeletal and movement-related functions |
| **1** | *cognitive_development* | **b1** | b117 Intellectual functions; b164 Higher-level cognitive functions |
| **1** | *language_development* | **d3 + b3** | d310–d330 Communicating—receiving/producing messages; b310 Voice functions; b320 Articulation functions |
| **1** | *social_emotional_development* | **d7 + b1** | d710 Basic interpersonal interactions; b152 Emotional functions |
| **1** | *parent_child_interaction* | **d7** | d760 Family relationships |
| **1** | *feeding_or_stool_transition* | **b5** | b510 Ingestion functions; b525 Defecation functions |
| **1** | *sleep_quality / sleep_or_behavior* | **b1** | b134 Sleep functions; b130 Energy and drive functions |
| **1** | *procedural_pain_or_stress* | **b2** | b280 Sensation of pain |
| **1** | *respiratory_or_cardiopulmonary* | **b4** | b440 Respiration functions; b410 Heart functions; b420 Blood pressure functions |
| **1** | *infection_or_ear_nose_throat* | **b4** | b435 Immunological system functions |
| **1** | *allergy_or_skin* | **b8 + b4** | b810 Protective functions of the skin; b435 Immunological system functions |
| **1** | *adverse_events / other* | **—** | Mixed systems; not elsewhere classified |

Step 2: ICF-CY codes aggregated into six outcome domains. Outcomes sharing the same core ICF-CY chapter were grouped into a common domain. For example, all outcomes coded to b5 (b530 and b540) that measured bilirubin-related indices were assigned to “Jaundice burden and bilirubin metabolism”; all outcomes involving standardised developmental assessments spanning d4 (Mobility), b1 (Mental functions), and b7 (Neuromusculoskeletal functions) were assigned to “Neurodevelopmental and motor function.” Outcomes reflecting physiological regulation, cardiorespiratory stability, pain, sensory function, sleep, and behavioural state were assigned to “Physiological regulation and safety”; binary developmental screening and classification outcomes were assigned to “Beneficial developmental classification.”

**Table S2.** Step 2 — Aggregation of ICF-CY codes into six outcome domains with rationale.

| **Step** | **Outcome Domain** | **Core ICF Dimension** | **Aggregation Rationale** |
| --- | --- | --- | --- |
| **2** | **1. Growth and anthropometry** | **b5 + s7** | Anthropometric parameters (weight, length, BMI, head circumference) reflect Body Structures (s7) regulated by metabolic functions (b530, chapter b5) |
| **2** | **2. Jaundice burden and bilirubin metabolism** | **b5** | Single biochemical pathway: bilirubin synthesis, conjugation, and clearance are metabolic functions (b540) within chapter b5 |
| **2** | **3. Neurodevelopmental and motor function** | **d4 + b1 + b7** | Standardised developmental scales assess Mobility (d4), Mental functions (b1: b117, b164), and Neuromusculoskeletal functions (b7: b710–b770) |
| **2** | **4. Feeding, stool transition, and digestion** | **b5** | Ingestion (b510) and defecation (b525) are both classified under chapter b5 (Digestive, metabolic and endocrine functions); key neonatal adaptation indicators |
| **2** | **5. Physiological regulation and safety** | **b4 + b2 + b1 + b8** | Multi-system regulation outcomes: cardiorespiratory (b4: b410, b420, b440), pain and sensory (b2: b280), sleep (b1: b134), immunological (b4: b435), and skin (b8: b810); treated as one integrated physiological regulation construct in neonatal care |
| **2** | **6. Beneficial developmental classification** | **b1 + d1** | Binary developmental screening and classification outcomes reflect whether an infant meets expected developmental criteria, integrating mental functions (b1) and learning (d1) |

Step 3: Data-driven allocation to meta-analysis vs. descriptive synthesis. This final step was determined by data availability rather than by theory. Domains containing two or more independent studies were eligible for random-effects meta-analysis using restricted maximum likelihood (REML) estimation. All six domains contained two or more independent studies and were eligible for random-effects meta-analysis. This criterion aligns with standard meta-analytic practice, which requires at least two independent effect sizes for between-study variance (τ²) estimation .

**Table S3.** Step 3 — Final allocation of outcome domains to synthesis method based on data availability.

| **Step** | **Outcome Domain** | **Studies**  **(k)** | **Effect-size Rows** | **Synthesis Method** |
| --- | --- | --- | --- | --- |
| **3** | **Neurodevelopmental and motor function** | 6 | 29 | Random-effects MA |
| **3** | **Growth and anthropometry** | 11 | 19 | Random-effects MA |
| **3** | **Physiological regulation and safety** | 2 | 5 | Random-effects MA |
| **3** | **Beneficial developmental classification** | 2 | 7 | Random-effects MA |
| **3** | **Jaundice burden and bilirubin metabolism** | 8 | 19 | Random-effects MA |
| **3** | **Feeding, stool transition, and digestion** | 4 | 8 | Random-effects MA |

*All six outcome domains met the minimum criterion of two or more independent studies and were included in random-effects meta-analysis using restricted maximum likelihood (REML) estimation.*

**3. Coding Reliability and Consensus**

Two reviewers (J.L.and G.L.) independently performed the ICF-CY coding. The initial agreement rate across the 15 outcome subcategories was documented, and all discrepancies were resolved through discussion. The final codebook, including the ICF-CY chapter assignments and the six-domain aggregation scheme, was reviewed and approved by the full review team before meta-analytic synthesis commenced.
