# Supplementary material for: Effects of formal aquatic activities on health and developmental outcomes in infants and toddlers younger than 36 months: a systematic review and meta-analysis
Source: Front Pediatr. 2026 Sep 30;14:1910257. doi: 10.3389/fped.2026.1910257 (PMC13662420; doi:10.3389/fped.2026.1910257)
Supplement: Supplementary file 2 [file Supplementaryfile2.docx]

***Supplementary Material 2***

***Figures***

***Figure S1 forest neurodevelopmental and motor function***

***Figure S2 funnel neurodevelopmental and motor function***

***Figure S3 forest growth and anthropometry***

***Figure S4 funnel growth and anthropometry***

***Figure S5 forest physiological regulation and safety***

***Figure S6 funnel physiological regulation and safety***

***Figure S7 forest beneficial developmental classification***

***Figure S8 funnel beneficial developmental classification***

***Figure S9 forest jaundice burden and bilirubin metabolism***

***Figure S10 funnel jaundice burden and bilirubin metabolism***

***Figure S11 forest feeding stool transition and digestion***

***Figure S12 funnel feeding stool transition and digestion***
