# Supplementary material for: Effects of formal aquatic activities on health and developmental outcomes in infants and toddlers younger than 36 months: a systematic review and meta-analysis
Source: Front Pediatr. 2026 Sep 30;14:1910257. doi: 10.3389/fped.2026.1910257 (PMC13662420; doi:10.3389/fped.2026.1910257)
Supplement: Supplementary file 3 [file Supplementaryfile3.docx]

**Supplementary Materials 3**

***Table S1 Sensitivity analysis for jaundice burden and bilirubin metabolism***

| **Analysis** | **SMD (95% CI); *I*^2^** | **P-value** |
| --- | --- | --- |
| Original model | 2.31 (1.37-3.26); *I*^2^=96.2% | <0.001 |
| Omit Feng 2025 | 2.10 (1.11-3.09); *I*^2^=96.2% | <0.001 |
| Omit Guo 2025 | 2.39 (1.31-3.47); *I*^2^=96.6% | <0.001 |
| Omit Chen 2025 | 2.26 (1.16-3.36); *I*^2^=96.3% | <0.001 |
| Omit Yu 2024 | 2.01 (1.17-2.85); *I*^2^=95.8% | <0.001 |
| Omit Wang 2013 | 2.47 (1.47-3.47); *I*^2^=96.0% | <0.001 |
| Omit Hou 2010 | 2.20 (1.17-3.23); *I*^2^=96.2% | <0.001 |
| Omit Liu 2009 | 2.48 (1.49-3.47); *I*^2^=96.0% | <0.001 |
| Omit Yan 2023 | 2.62 (1.68-3.57); *I*^2^=95.7% | <0.001 |

***Table S2 Subgroup analyses for jaundice burden and bilirubin metabolism***

| **Subgroup analysis** | **SMD (95% CI); *I^2^*** | **P-value** |
| --- | --- | --- |
| Outcome type - Bilirubin or jaundice index level | 2.33 (1.32-3.33); ***I*^2^**=96.6% | <0.001 |
| Outcome type - Jaundice duration/regression time | 1.90 (1.19-2.61); ***I*^2^**=78.9% | <0.001 |
| Population - Healthy/non-clinical newborns | 1.67 (0.01-3.33); ***I*^2^**=99.3% | 0.048 |
| Population - Jaundice/hyperbilirubinemia clinical samples | 2.58 (1.41-3.76); ***I*^2^**=95.9% | <0.001 |
| Intervention - Aquatic exposure plus touch/rehabilitation/bundled care | 1.86 (0.98-2.75); ***I*^2^**=95.6% | <0.001 |
| Intervention - Aquatic exposure without added touch | 3.76 (2.94-4.58); ***I*^2^**=74.6% | <0.001 |

***Table S3 study characteristics and literature DOI hyperlink***

| **Study id** | **Author year** | **Age or gestational age description** | **Sex distribution extracted** | **Final effects contributed** | **Included in final meta dataset** | **Final analysis categories** | **Final effect measures** |
| --- | --- | --- | --- | --- | --- | --- | --- |
| [Dias_2013](https://doi.org/10.2466/10.25.pms.117x30z2) | Dias et al. 2013 | Healthy full-term infants aged 7 to 9 months | not reported in current extraction pass | 0 | no |  |  |
| [Leo_2022](https://doi.org/10.3390/ijerph19159262) | Leo et al. 2022 | Healthy full-term infants; post-intervention analytic sample n=28 | Experimental aquatic group: 9 males, 5 females; control group: 8 males, 6 females. | 8 | yes | Neurodevelopmental and motor function (SMD) | SMD |
| [Zhao_2005](https://doi.org/10.1080/14767050400028782) | Zhao et al. 2005 | Normal term newborns during birth hospitalization | not reported in current extraction pass | 2 | yes | Growth and anthropometry (SMD) | SMD |
| [McManus_2007](https://doi.org/10.1097/pep.0b013e3181575190) | McManus and Kotelchuck 2007 | Infants and toddlers aged 6 to 30 months with delayed functional mobility | Aquatic therapy group: 9 males, 6 females; control group: 9 males, 11 females. | 1 | yes | Neurodevelopmental and motor function (SMD) | SMD |
| [Araujo_2023](https://doi.org/10.1080/03004430.2022.2048828) | Araujo et al. 2023 | Control group mean age 10.37 +/- 2.92 months; intervention group mean age 11.53 +/- 3.35 months. | Control group: 17 males (45.95%); intervention group: 15 males (62.50%). | 3 | yes | Beneficial developmental classification (RR) | RR_log |
| [Franca_2026](https://doi.org/10.1080/09593985.2026.2621956) | Franca et al. 2026 | Mean gestational age at birth 30.88 +/- 1.86 weeks; mean birth weight 1451.91 +/- 378.61 g. | Overall sample: 52.9% girls. | 1 | yes | Growth and anthropometry (SMD) | SMD |
| [Tedesco_2021](https://doi.org/10.1080/09593985.2021.1926025) | Tedesco et al. 2021 | Hospitalized stable preterm newborns with low birth weight | not reported in current extraction pass | 2 | yes | Growth and anthropometry (SMD); Physiological regulation and safety (SMD) | SMD |
| [Feng_2025](https://doi.org/10.16344/j.cnki.10-1669/r4.2025.06.047) | Feng 2025 | Neonates with neonatal hyperbilirubinemia | Control group: 20 males, 15 females; observation group: 18 males, 17 females. | 2 | yes | Jaundice burden and bilirubin metabolism (SMD; lower favorable) | SMD |
| [Zhang_2025](https://doi.org/10.12385/j.issn.2096-1278(2025)01-0151-03) | Zhang et al. 2025 | Intervention group age 1-28 days, mean 14.62 +/- 3.43 days; control group age 1-27 days, mean 13.83 +/- 3.04 days. | Intervention group: 28 males, 26 females; control group: 30 males, 24 females. | 9 | yes | Growth and anthropometry (SMD); Neurodevelopmental and motor function (SMD) | SMD |
| [Pan_2025](https://doi.org/10.3969/j.issn.2097-115X.2025.12.032) | Pan et al. 2025 | Gestational age 37-42 weeks; observation group mean 39.21 +/- 1.08 weeks; control group mean 39.40 +/- 1.13 weeks. | Observation group: 22 males, 24 females; control group: 25 males, 21 females. | 2 | yes | Growth and anthropometry (SMD) | SMD |
| [Guo_2025](https://doi.org/10.14164/j.cnki.cn11-5581/r.2025.12.048) | Guo 2025 | Control group age 2-25 days, mean 13.54 +/- 3.63 days; observation group age 2-24 days, mean 13.23 +/- 3.76 days. | Control group: 17 males, 13 females; observation group: 18 males, 12 females. | 8 | yes | Jaundice burden and bilirubin metabolism (SMD; lower favorable); Neurodevelopmental and motor function (SMD) | SMD |
| [Chen_2025](https://doi.org/10.20256/j.cnki.zgdxbl.20250414.001) | Chen 2025 | Control group gestational age 36-40 weeks, mean 38.62 +/- 0.37 weeks; observation group gestational age 37-41 weeks. | Control group: 18 males, 21 females; observation group: 17 males, 22 females. | 6 | yes | Growth and anthropometry (SMD); Jaundice burden and bilirubin metabolism (SMD; lower favorable) | SMD |
| [Yu_2024](https://doi.org/10.3969/ j.issn.672-0369.2024.15.026) | Yu et al. 2024 | Term neonates with bilirubin encephalopathy; control gestational age 38-41 weeks. | Control group: 24 males, 22 females; observation group: 23 males, 23 females. | 3 | yes | Jaundice burden and bilirubin metabolism (SMD; lower favorable) | SMD |
| [Wu_2020](https://doi.org/10.19829/j.zgfybj.issn.1001-4411.2020.24.043.) | Wu et al. 2020 | Observation group gestational age 39.38 +/- 1.39 weeks; control group gestational age 39.42 +/- 1.42 weeks. | Observation group: 30 males, 22 females; control group: 29 males, 23 females. | 0 | no |  |  |
| [Cao_2016](https://doi.org/10.19460/j.cnki.0253-3685.2016.17.035) | Cao 2016 | Preterm infants | Overall sample: 54 males, 32 females. | 4 | yes | Beneficial developmental classification (RR) | RR_log |
| [Wang_2013](https://doi.org/10.3969/j.issn.1672-9676.2013.01.003) | Wang et al. 2013 | Healthy term newborns | not reported in current extraction pass | 5 | yes | Feeding, stool transition, and digestion (SMD; direction aligned); Growth and anthropometry (SMD); Jaundice burden and bilirubin metabolism (SMD; lower favorable) | SMD |
| [Huang_2012](https://doi.org/10.3969/j.issn.1672-9676.2012.19.007) | Huang 2012 | Age 1-24 days, mean 15.5 +/- 4.5 days; gestational age 39.6 +/- 5.2 weeks. | Overall sample: 52 males, 48 females. | 8 | yes | Growth and anthropometry (SMD); Neurodevelopmental and motor function (SMD) | SMD |
| [Hou_2010](https://kns-cnki-net-443.v.bsu.edu.cn/kcms2/article/abstract?v=wGRGwcz8A0cr3NMfwsAJHwVWQsXkXFXqa_8ZgLTbH9kTUtItCyCC1I7DL8xc7H8sMdmDrCzTzCrkaHk2v1XSJs4u15yZeRMoAqSqVoyJ5UwGMztDFuPZOUGG6bwwhr7xRFiMp-DcYinpkbTfaFmSzAG4ic7KdjRCQnRny8jo4Yy4wewlnOdytA==&uniplatf) | Hou and Zhang 2010 | Normal term newborns | not reported in current extraction pass | 1 | yes | Jaundice burden and bilirubin metabolism (SMD; lower favorable) | SMD |
| [Liu_2009](https://kns-cnki-net-443.v.bsu.edu.cn/kcms2/article/abstract?v=wGRGwcz8A0dvd3853KiDNQKsxnJP82c6JpnscT8cXGylEhPjugnUd6zYyknclMbi4VQL9yxQ0Zwc3RFkMm7DK5klazyu1j00zhk1ghD8zNOKwPtZom-K1djNR-E5vE8gaXzOqngvJKpU2QIQkx0AdTTqcYoOuOC0&uniplatform=NZKPT) | Liu 2009 | Cesarean-born newborns | not reported in current extraction pass | 5 | yes | Feeding, stool transition, and digestion (SMD; direction aligned); Growth and anthropometry (SMD); Jaundice burden and bilirubin metabolism (SMD; lower favorable) | SMD |
| [Huang_2006](https://doi.org/10.16252/j.cnki.issn1004-0501-2006.06.054) | Huang et al. 2006 | Newborns with physiological weight loss | not reported in current extraction pass | 1 | yes | Growth and anthropometry (SMD) | SMD |
| [Piao_2005](https://kns-cnki-net-443.v.bsu.edu.cn/kcms2/article/abstract?v=wGRGwcz8A0eB7QNhMgNdpi63GWWzkJqr2HbsMWPxFaq4gK0py6CRuyMywvqwr0-YaoO9hD7TSNe2w7dP-UUbEAY6Y23-_74TmP6gcs7vl3hYrdbZXccOmAW6bY-u2KhmniTCbnAfvAsdu8ZJPHcSIP9t_0mv7eUf&uniplatform=NZKPT) | Piao et al. 2005 | Normal newborns and 2-month infants | not reported in current extraction pass | 0 | no |  |  |
| [Wen_2005](https://kns-cnki-net-443.v.bsu.edu.cn/kcms2/article/abstract?v=wGRGwcz8A0dM0xTMRdqPo6Tgp31EyQwGBruBPVlP38EqkyYNDmKu3KpgN7wKWEZv4UJ5xxz1mZ0eaAVIYqfeJ1cToqaBApBLpenXO9tjNIt0Qss00BYkDmT-HoHyMt3ubw3oNnAnKnKIx-zFAJim7vM84Z0wygpx&uniplatform=NZKPT) | Wen et al. 2005 | Normal newborns during hospitalization | not reported in current extraction pass | 3 | yes | Feeding, stool transition, and digestion (SMD; direction aligned); Growth and anthropometry (SMD) | SMD |
| [Zhong_2005](https://kns.cnki.net/kcms2/article/abstract?v=wGRGwcz8A0dZJwM7a3RhIGj5-sl_UefrwbnNeRVjQn0bdwD5TUraEblIcdaMfAJfPeCNnKKzurrUFAc3h12NOdoUvJ-MPauHHQaxjIbyJJihHrIpVkjV7Uq4KpweqCnMV0r4ppKv1f2_WtdYr_bIsPGJdjsnhhVx&uniplatform=NZKPT) | Zhong and Liang 2005 | Overall sample: 30 full-term, 27 preterm, and 3 post-term neonates; day age categories up to 7 days. | Overall sample: 34 males, 26 females. | 4 | yes | Physiological regulation and safety (SMD) | SMD |
| [Li_2025](https://kns-cnki-net-443.v.bsu.edu.cn/kcms2/article/abstract?v=wGRGwcz8A0cNuS0MpsRvAx28VOzeT5V5fjV29CJS-FWsMCbly7ofiR-oEOtJ1e8aJiBNt9OTO4HV7ZK9zZlt1oLYUXJpSlWQ9D0x2o4O2nR9Nbm2fnZ85KKtUuELdR2eZayEMkLy9IS_UoHq18rYaMDJOk9q3MOwdzTQ9lz7lmOwGfpaZp_jlA==&uniplatf) | Li 2025 | Neonates with neurological developmental delay | not reported in current extraction pass | 3 | yes | Neurodevelopmental and motor function (SMD) | SMD |
| [Yan_2023](https://kns-cnki-net-443.v.bsu.edu.cn/kcms2/article/abstract?v=wGRGwcz8A0ersDNL2UsP8ElJsMiJ-n_fKow7yJrpO0w36mm9PVNjA-ielAa1lsDl-LpQs2aGJl2H5KHxOkDWQKKMVF0-xo_0-myMbes0cZOmNGBQfkoyoDt8iQqxtjeDHFHtxhWBeWZjoqd8ZAJlXoSpMRdX5wqO1XPmpt2yufg=&uniplatform=NZKPT) | Yan et al. 2023 | Routine group age 2-20 days, mean 12.12 +/- 3.91 days; combined group age 2-20 days, mean 12.32 +/- 4.01 days. | Routine group: 23 males, 18 females; combined group: 21 males, 20 females. | 6 | yes | Jaundice burden and bilirubin metabolism (SMD; lower favorable); Feeding, stool transition, and digestion (SMD; direction aligned) | SMD |

***DOI and web addresses are displayed as hyperlinks in the study id column.***
